# High rates of retention and viral suppression in the scale-up of antiretroviral therapy adherence clubs in Cape Town, South Africa

**DOI:** 10.7448/IAS.20.5.21649

**Published:** 2017-07-21

**Authors:** Priscilla Ruvimbo Tsondai, Lynne Susan Wilkinson, Anna Grimsrud, Precious Thembekile Mdlalo, Angelica Ullauri, Andrew Boulle

**Affiliations:** ^a^ School of Public Health and Family Medicine, University Of Cape Town, Cape Town, South Africa; ^b^ Médecins Sans Frontières, Cape Town, South Africa; ^c^ International AIDS Society, Cape Town, Khayelitsha, South Africa; ^d^ Department of Health, Provincial Government of the Western Cape, Cape Town, South Africa

**Keywords:** HIV, antiretroviral therapy, models of care, adherence club, retention, program outcomes

## Abstract

**Introduction**: Increasingly, there is a need for health authority scale up of successfully piloted differentiated models of antiretroviral therapy (ART) delivery. However, there is a paucity of evidence on system-wide outcomes after scale-up. In the Cape Town health district, stable adult patients were referred to adherence clubs (ACs) – a group model of ART delivery with five visits per year. By the end of March 2015, over 32,000 ART patients were in an AC. We describe patient outcomes of a representative sample of AC patients during this scale-up.

**Methods**: Patients enrolled in an AC at non-research supported sites between 2011 and 2014 were eligible for analysis. We sampled 10% of ACs (*n* = 100) in quintets proportional to the number of ACs at each facility, linking each patient to city-wide laboratory and service access data to validate retention and virologic outcomes. We digitized registers and used competing risks regression and cross-sectional methods to estimate outcomes: mortality, transfers, loss to follow-up (LTFU) and viral load suppression (≤400 copies/mL). Predictors of LTFU and viral rebound were assessed using Cox proportional hazards models.

**Results**: Of the 3216 adults contributing 4019 person years of follow-up (89% in an AC, median 1.1 years), 70% were women. Retention was 95.2% (95% CI, 94.0-96.4) at 12 months and 89.3% (95% CI, 87.1-91.4) at 24 months after AC enrolment. In the 13 months prior to analysis closure, 88.1% of patients had viral load assessments and of those, viral loads ≤400 copies/mL were found in 97.2% (95% CI, 96.5-97.8) of patients. Risk of LTFU was higher in younger patients and in patients accessing ART from facilities with larger ART cohorts. Risk of viral rebound was higher in younger patients, those that had been on ART for longer and patients that had never sent a buddy to collect their medication.

**Conclusions**: This is the first analysis reporting patient outcomes after health authorities scaled-up a differentiated care model across a high burden district. The findings provide substantial reassurance that stable patients on long-term ART can safely be offered care options, which are more convenient to patients and less burdensome to services.

## Introduction

By the end of 2015, UNAIDS estimated that over 17 million people were receiving antiretroviral therapy (ART) globally [1]. This number is expected to further increase as national programs adopt the new “treat all” WHO guidelines, recommending ART for all HIV-infected people [2]. Given the importance of ensuring patients initiated on ART are retained in care, it is imperative that ART services are equipped not only to accommodate this anticipated increase in the number of people receiving treatment, but also to ensure quality care for those who are already on treatment. One such way is through a differentiated model of ART delivery.

ART adherence clubs (ACs) are a health care worker-managed group model designed for stable patients on treatment. ART refill visits are separated from clinical consultations thereby reducing the frequency of clinic visits, decongesting the clinics and providing an efficient ART delivery system [3]. A pilot study conducted by Médecins Sans Frontières (MSF) in Cape Town, South Africa in 2007 showed that retention among patients managed within ACs was 97% compared with 85% among patients who qualified for club participation but remained in routine care over 40 months. It further demonstrated that ACs reduced loss-to-follow-up (LTFU) and virologic rebound by 57% and 67% respectively [4]. Following this success, ACs were adopted by the Western Cape and the City of Cape Town Departments of Health in 2011 and implemented across the entire Cape Town health district. This scale-up has been described in detail previously [5]. As of March 2015, 1308 ACs were running from 55 of the 70 ART facilities within the district. Approximately 25% of all ART patients in the district, or over 32,000 patients, were being supported within these ACs across the district [5]. However, no data exists on patient outcomes following the scale up of this model of care. This study therefore, describes and explores possible predictors of LTFU and viral rebound for a representative sample of patients receiving their ART within ACs in Cape Town, South Africa.

## Methods

We conducted a retrospective observational cohort study of patients enrolled in the AC model within the Cape Town health district between January 2011 and December 2014.

### Setting

The Cape Town health district is one of the six districts within the Western Cape Province of South Africa, with an estimated population of approximately 3.7 million over an area of 2461 km^2^ [6]. The antenatal HIV prevalence in the district was 21.7% in 2013, compared to a national average of 29.7% [7]. ART has been provided within the district since 2001, initially as a pilot project by MSF and the Western Cape Government, and subsequently through a range of additional partnerships and with formal routine availability in government services from 2004.

### ART adherence clubs

ART ACs are a group model for stable patients on ART and have been described in detail previously [3]. In brief, each AC has approximately 25-30 patients who meet five times a year either within the health care facility or at a community venue for a brief symptom screen, group discussion and to receive their pre-packed ART supply. Participation in an AC is voluntary for “stable” patients defined before March 2015 as being on ART >12 months with two consecutive suppressed viral loads (<400 copies/mL) and thereafter as being on ART for >6 months, virally suppressed (<400 copies/mL) at the last viral load assessment and having no other condition requiring more frequent clinical consultation. AC meetings are mostly facilitated by lay health care workers with support from clinical staff. Four months after joining an AC and annually thereafter, patients have their blood drawn for viral load assessment. At the club meeting following the blood assessments, patients meet a clinician for a clinical consultation including a review of their viral load result. Patients are able to send a treatment partner known as a “buddy” to collect their medication at alternate club meetings. Once a patient develops symptoms suggestive of ill-health or requires more frequent adherence or clinical follow up (including when the patient’s viral load is >400 copies/mL), they are referred back to clinician-led standard-of-care at the facility. Each AC has a paper-based register in which attendance, weight and referrals are recorded at each visit. This data are then captured into the facility’s electronic monitoring system by the clinic data clerks [3,8].

### Sampling and data collection

All ACs within the Cape Town health district at primary healthcare facilities without substantial operational research support for differentiated care implementation were eligible for this analysis. As of December 2014, 976 ACs were eligible. A weighted random sample, with each club as the sampling unit was used to sample approximately 10% of eligible ACs in order for the sample to be representative of all AC patients in the district. Twenty random selection points (ACs) were generated and for each club selected, data was extracted from the selected club, the two clubs before and the two clubs after. One hundred ACs from 15 health care facilities were selected for inclusion and of these, replacement sampling with sequential ACs was done for 6 ACs due to their registers not being available. Photographic images of the selected ACs registers were taken at the health care facility. Data from these images were abstracted and entered using double data entry and validation, into the Research Electronic Data Capture (REDCap) database hosted at the University of Cape Town [9]. Patient clinic folders were then reviewed and data on clinic visits, ART refills and viral loads were abstracted for all patients who had either defaulted from the ACs or been referred back to the clinic. In addition, data on formal service contacts and viral load results were extracted from the Provincial Health Data Centre which houses province-wide routine data, to validate retention and virologic outcomes.

### Statistical analysis

All patients within the sampled ACs were eligible for this analysis if they had joined an AC between January 2011 and December 2014 and had attended at least one AC meeting. Patients entered the analysis at their first club meeting and exited at the date of analysis closure (31 December 2014), date of outcome or date of censoring. The outcomes of interest in this analysis were LTFU and viral rebound. LTFU was defined as having no contact with an AC or clinic in the 6 months following analysis closure (1 January to 30 June 2015) and was determined to have happened on the date of last contact with the service. Viral rebound was defined as the first viral load result >400 copies/mL after enrolment into an AC.

Patient characteristics at enrolment into a club (age, sex, time on ART, year of AC enrolment) were summarized using medians and interquartile ranges (IQRs) for continuous variables and proportions for categorical variables. The proportion of patients with viral load assessments performed and the associated viral load results for the first three viral load assessments after enrolment into a club (months 4, 16 and 28) were summarized using percentages with binomial 95% confidence intervals (CIs). The viral load measurement for each assessment point was defined as the closest measure within a 12 month window. In addition, a cross sectional analysis was done of the proportion of patients with a viral load assessment and the corresponding viral load results within 13 months prior to study closure (November 2013-December 2014). Competing risks regression was used to estimate the cumulative incidence for LTFU, transfer out (TFO) and mortality which were then used to calculate the corresponding cumulative retention. We used competing risks to calculate the cumulative incidences as our outcomes are mutually exclusive [10,11]. Predictors of being LTFU and experiencing viral rebound were then assessed using univariable and multivariable Cox proportional hazards models, adjusting for the baseline covariates (age, sex, time on ART, having ever sent a buddy, number of clubs at facility per 1000 patients on ART, total number of patients on ART at facility). Results are presented as hazard ratios (HR) and adjusted hazards ratios (aHR) with 95% CIs. Data were analysed using Stata 13.0 (STATA Corporation, College Station, TX, USA).

### Ethics

This study was approved by the Human Research Ethics Committee (HREC) in the Faculty of Health Sciences at the University Of Cape Town (HREC REF 535 /2015) as well as the Provincial Government of the Western Cape and the City of Cape Town Departments of Health. Informed consent was not sought from individual patients as this study was a retrospective cohort analysis of routinely collected data.

## Results

A total of 3775 patients were recorded in all the selected AC registers and of these, 532 patients who enrolled after analysis closure (after 31 December 2014) and 27 patients who had never attended a club meeting were excluded. The 3216 patients included in this analysis contributed 4019 person-years of follow up (median 1.1 years; Interquartile range (IQR), 0.7-1.6), 89% of which was spent in ACs. At AC enrolment; median age was 36.4 (IQR, 31.5-41.6) years, 70% were female and patients had been on ART for a median 2.8 (IQR, 1.8-4.4) years (Table 1). The majority of patients had joined an AC in the year 2014 (*n* = 1399), with only 591 having joined in the years 2011 and 2012 (18.4%).

**Table 1.** Description of the sampled adherence club patients (*N* = 3216)

Characteristics	Patients in adherence clubs, *n* (%)
Age at club start (years)	3210 (99.8)
16-24	119 (3.7)
25-34	1265 (39.4)
35-44	1323 (41.2)
≥45	503 (15.7)
Median (IQR)	36.4 (31.5-41.6)
Sex	3202 (99.6)
Female	2254 (70.4)
Male	948 (29.6)
Year of club start	3216 (100)
2011	113 (3.5)
2012	478 (14.9)
2013	1226 (38.1)
2014	1399 (43.5)
Duration on ART at club start (years)	3112 (96.8)
<2	925 (29.7)
2-4	1247 (40.1)
>4	940 (30.2)
Median (IQR)	2.8 (1.8-4.4)

By analysis closure, 4 patients (0.1%) had died, 82 patients (2.6%) had transferred care and 135 (4.2%) patients were LTFU (Table 2), with no differences observed between males and females (*p* = 0.289). One hundred and forty-five patients initially classified as LTFU when using data from the registers and patient folders, were found to still be in care either at their original facilities (*n* = 138) or silently transferred to a different facility (*n* = 7) after linking to service access and laboratory data. Cross-sectional retention at study closure was 88.8% using data from the registers and patient clinic folders and 93.1% after database linkage. One-third of patients (*n* = 1084, 33.7%) had sent a buddy to collect their medication at least once during the analysis period.

**Table 2.** Cross-sectional outcomes at analysis closure for adherence club patients

	Before database linkage^a^		After database linkage^b^	
	*n*	(%)	*N*	(%)
Retained	2857	(88.8)	2995	(93.1)
Retained continuously in club	2497	(77.6)	2497	(77.6)
Retained in club with facility based care	36	(1.1)	36	(1.1)
Retained at facility	324	(10.1)	462^c^	(14.4)
Transferred out (TFO)	75	(2.3)	82	(2.6)
Formal TFO	75	(2.3)	75	(2.3)
Silent TFO	–	–	7	(0.2)
Lost to follow-up (LTFU)	280	(8.7)	135	(4.2)
LTFU – last seen in club	219	(6.8)	46	(1.4)
LTFU – last seen at facility	61	(1.9)	89	(2.8)
Died	4	(0.1)	4	(0.1)

^a^Using data from adherence club registers and patient clinic folders within facilities. ^b^After database linkage of patients classified as LTFU to city wide laboratory and service access data. ^c^Patients initially classified as LTFU due to missing clinic folders but found to still be in care at same facility after database linkage.

**Figure 1.**
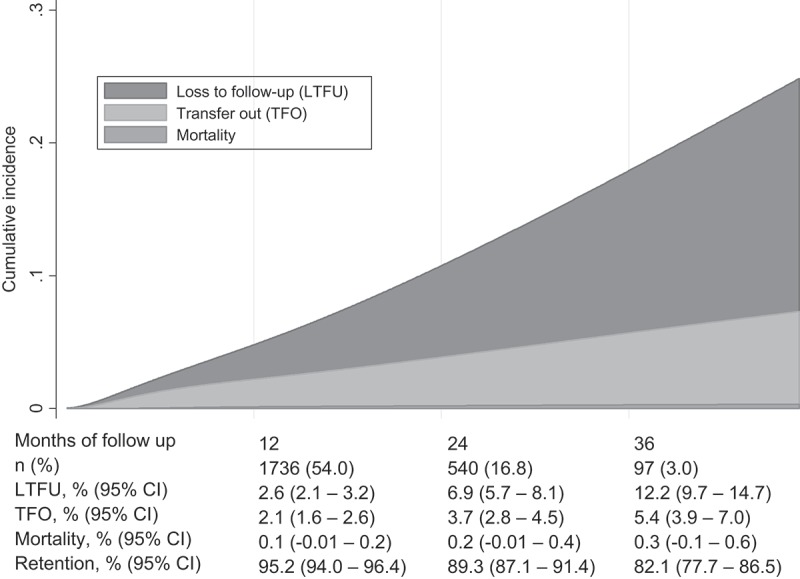
Stacked cumulative incidence of mortality, transfers and loss to follow up.

Cumulative incidence of LTFU, TFO and deaths was 2.6% (95% CI, 2.1-3.2), 2.1% (95% CI, 1.6-2.6) and 0.1% (95% CI, −0.01 to 0.2) at 12 months, rising to 12.2% (95% CI, 9.7-14.7), 5.4% (95% CI, 3.9-7.0) and 0.3% (95% CI, −0.1 to 0.6) at 36 months after AC enrolment, respectively (Figure 1). Cumulative retention was therefore, 95.2% (95% CI, 94.0-96.4) after 12 months, 89.3% (95% CI, 87.1-91.4) after 24 months and 82.1% (95% CI, 77.7-86.5) after 36 months of follow-up.

Viral load assessments at month 4, 16 and 28 were performed in 86.5%, 84.7% and 79.7% of patients, respectively (Table 3). Of those that had a viral load done, viral load ≤400 copies/mL were found in 96.9% (95% CI, 96.2-97.6) at month 4, 95.7% (95% CI, 94.6-96.7) at month 16 and 94.1% (95% CI, 91.6-96.0) at month 28 after joining an AC. In a cross-sectional analysis of viral load assessments within the 13 months before analysis closure, 88.1% (95% CI, 86.9-89.2) of patients had a viral load test performed and 97.2% (95% CI, 96.5-97.8) of those patients had a viral load ≤400 copies/mL (results not shown).

**Table 3.** Viral load assessments and viral suppression at months 4, 16 and 28 after adherence club enrolment

Months of follow up	4	16	28
Patients followed (*n*)	3216	1846	615
Viral loads done, *n* (%)	2782 (86.5)	1563 (84.7)	490 (79.7)
Results (copies/mL), *n* (%)			
≤400	2697 (96.9)	1496 (95.7)	461 (94.1)
401-1000	24 (0.9)	11 (0.7)	4 (0.8)
>1000	61 (2.2)	56 (3.6)	25 (5.1)

In proportional hazards models, the hazard of LTFU was higher in patients aged 16-24 years and in patients receiving ART at facilities with larger ART cohorts (Table 4). These associations persisted in adjusted models. There was an increased risk of LTFU in those aged 16-24 years (aHR 2.41, 95% CI, 1.10-5.23), and in those aged 25-34 years (aHR 1.55, 95% CI, 1.03-2.33) compared to patients aged 35-44 years. In patients receiving ART from facilities with larger ART cohorts, the aHR was 1.32 for every 1000 patients in care on ART (95% CI, 1.11-1.58). No differences in the risk of LTFU were found by sex, duration on ART, whether patients sent a buddy to collect their medication or not, or by the proportion of patients in care who were in the club model represented by the number of clubs available at a facility per 1000 patients on ART.

**Table 4.** Relative hazard of LTFU and viral rebound among adherence club patients

Characteristic	LTFU		Viral rebound^a^	
	Univariate HR (95% CI)	aHR (95% CI) (n = 3106)	Univariate HR (95% CI)	aHR (95% CI) (n = 3106)
Age at AC enrolment (years)				
16-24	2.16 (1.06-4.40)	2.41 (1.10-5.23)	1.60 (0.68-3.76)	1.52 (0.59-3.95)
25-34	1.37 (0.93-2.00)	1.55 (1.03-2.33)	1.64 (1.11-2.41)	1.74 (1.17-2.59)
35-44	1.0 (ref)	1.0 (ref)	1.0 (ref)	1.0 (ref)
≥ 45	0.99 (0.58-1.69)	1.04 (0.60-1.82)	0.70 (0.37-1.32)	0.69 (0.36-1.31)
Sex				
Male	0.99 (0.68-1.44)	1.13 (0.77-1.68)	0.77 (0.51-1.15)	0.94 (0.62-1.43)
Duration on ART at AC				
enrolment (years)	0.97 (0.88-1.07)	0.98 (0.89-1.09)	1.07 (0.98-1.17)	1.12 (1.03-1.23)
Ever sent a buddy				
Yes	0.75 (0.52-1.07)	0.79 (0.55-1.14)	0.63 (0.43-0.92)	0.63 (0.43-0.93)
Number of clubs at facility/1000 patients	1.01 (0.93-1.09)	1.02 (0.93-1.11)	0.96 (0.89-1.03)	0.94 (0.87-1.02)
Number of patients on ART in facility/1000	1.34 (1.13-1.59)	1.32 (1.11-1.58)	0.99 (0.81-1.20)	0.97 (0.79-1.18)

HR: hazard ratio, aHR: adjusted hazard ratio, CI: confidence interval, AC: adherence club, ART: antiretroviral therapy. ^a^Viral rebound defined as the first viral load >400 copies/mL after enrolment into an AC.

Patients aged 25-34 years also experienced an increased risk of viral rebound (Table 4). In the adjusted model, the risk was higher in patients aged 25-34 compared to those aged 35-44 years (aHR 1.74, 95% CI, 1.17-2.59). The risk of viral rebound was higher in patients who had been on ART for longer (aHR for each additional year on ART of 1.12, 95% CI, 1.03-1.23). Patients who sent a buddy to collect their medication at least once had a 37% decrease in the risk of experiencing a viral rebound (aHR 0.63, 95% CI, 0.43-0.93).

## Discussion

To our knowledge, this is the first analysis reporting patient outcomes after ART ACs were scaled-up by health authorities across a high burden district. This study demonstrates that ART ACs have high levels of retention and viral suppression for stable patients on ART, even at scale.

There is a growing body of evidence on the safety and effectiveness of differentiated models of ART delivery [4,12–19]. Studies from South Africa, Mozambique, Uganda and Kenya have all shown that differentiated care models used to deliver ART can achieve comparable or even higher rates of retention and viral suppression than the traditional facility-based clinician-led model of care. These all provide re-assurance that stable patients can be safely shifted to less intensive follow up without compromising their clinical outcomes, making life long disease management more efficient for patients and freeing up valuable clinician time for patients requiring more intense follow-up or those being newly initiated.

We observed an increased risk of LTFU and viral rebound in younger patients; a finding that has been demonstrated in other studies [14,20–24]. Poor outcomes for young people are not limited to models of differentiated care or HIV. Young people face specific challenges in managing chronic health issues which may impact on their adherence to medication schedules or clinic visits, putting them at risk of disengaging from care. Further issues then arise for these patients when they have been classified as stable on ART while transitioning through these difficult adolescent and young adult phases. HIV care programs therefore, need to acknowledge the challenges among this group of patients and ensure that care meets their needs and expectations. Of interest is that poorer retention outcomes as well as increased viral rebound were also experienced in the 25-34 year olds, a group not generally considered as being at increased risk and requiring age-directed health care delivery [20,22,23].

We found that the risk of viral rebound increased with each additional year a patient had been on ART before joining an AC and that the risk of being LTFU increased with each additional 1000 patients added to the ART cohort in the facility. We anticipate an expansion of ART treatment services in resource-limited settings as countries adopt the “treat all” recommendations [2]. Our findings highlight the need for measures to be put in place to guard against deteriorating quality of care within these types of models, as is the case with traditional facility based clinician-led models, as treatment cohorts expand and as ART cohorts mature.

Sending a buddy to collect an ART refill reduced the risk of viral rebound and did change the risk of LTFU. Patients who are unable to attend services in person and have the agency to send someone to collect their medicine may differ from other patients in unmeasured ways, resulting in residual confounding. Nevertheless this finding supports a move towards both extending the ART refill interval beyond two or three months and encouraging patients to make use of the buddy mechanism if they cannot attend. Allowing patients to still have access to their medications even when they themselves are physically unable to visit a health care facility may contribute to patients being more adherent.

Worse clinical outcomes have been consistently observed among men [23,25–31], even when accessing ART through a differentiated model of care [12]. In our analysis, no difference in outcomes was found by sex, a finding which has also been demonstrated in some community-based models of ART delivery [14,24]. Men who opt for accessing their care through the AC model may be systematically different as not only were they doing well in routine care but by joining an AC, they are consenting to be part of a group model with the majority of members being women.

There are several strengths to this study. Our data are from routine services – ACs run within facilities without substantial research or external technical support, across an entire district. These results could, therefore be generalizable to many health care service facilities. In this district, patients have access to routine viral load monitoring and therefore we were able to report both programmatic and virological outcomes. Also, due to the presence of unique identifiers in this district, our study was able to link patients across different services enabling us to differentiate true LTFU from silent transfers and also to report more complete viral load outcomes.

Some limitations need to be considered in the interpretation of these findings. Some old registers were not available during data collection which could have resulted in the exclusion of patients in the initial phase of ACs scale-up who might have defaulted from care. Our analysis was limited to the variables routinely collected in the AC registers and therefore we could not assess the impact of other key variables such as disclosure and ART regimen on patient outcomes. The follow-up time in this analysis was limited and there were relatively small numbers of patients with sufficient long term potential follow-up to assess long term outcomes as nearly half of the patients joined an AC in the same year as the database closure. In addition, only a small number of patients aged 16-24 years were included in our analysis limiting our ability to comprehensively make conclusions on the outcomes for this age group. We acknowledge that LTFU represents all patients with an unknown outcome and that our study was not designed to compare outcomes between patients accessing ART within ACs and those retained in the clinician-led standard of care.

Differentiated models of care are an inevitable necessity. Growth of and changing eligibility for ART services requires models that provide options of varying formal health service intensity in order to provide patient-centred quality services for all patients. The AC model has already been adopted as South African [32] national policy and similar models are in the national guidelines of Swaziland [33] and Zimbabwe [34]. With our findings of good outcomes at scale, there is increased evidence for adoption. Further, these findings support patient responsive ART services, extending the ART refill interval and reducing the need for clinical review consultations to once a year with access to routine viral load monitoring. Though data used in this analysis was from 2014 with follow up until June 2015, it is relevant for current policies with the anticipated expansion of ART programs as a differentiated model of care that has produced good patient outcomes at scale. Further research is needed to understand patient expectations and challenges within differentiated care models. In addition, health system research examining the process issues related to establishing, extending and maintaining the model would be beneficial to health policy makers.

In conclusion, these findings provide substantial reassurance that the AC model supports good patient outcomes at scale for stable patients on long-term ART.
